# Predictive Factors and Clinical Prediction Score for Serious Intracranial Causes in Acute Nontraumatic Headache at an Emergency Department

**DOI:** 10.1155/2019/4267825

**Published:** 2019-10-31

**Authors:** Siriwimon Tantarattanapong, Lalita Chalongkulasak

**Affiliations:** Department of Emergency Medicine, Songklanagarind Hospital, Faculty of Medicine, Prince of Songkla University, Hat Yai, Songkhla 90110, Thailand

## Abstract

**Purpose:**

The objectives of this study were to investigate the predictive factors and develop a clinical prediction score to identify serious intracranial causes in acute nontraumatic headache (NTH).

**Methods:**

A retrospective chart review study was conducted from 2013 to 2018 in acute NTH patients who visited the emergency department. The patients were divided into serious intracranial headache and nonserious intracranial headache groups. The two groups were compared in regard to the baseline characteristics, clinical presentation, physical examination, investigation, and diagnosis. The significant factors to predict a serious intracranial cause were examined using a multivariate logistic regression model. The coefficients from the multivariate logistic regression were used to plot the receiver operating characteristic curve to develop a clinical prediction score.

**Results:**

From 2,372 patients, 454 met the inclusion criteria. Of the 454 patients with acute NTH, 88 (19.4%) patients were serious intracranial cause. The seven significant factors that predicted serious intracranial cause were abrupt onset (odds ratio (OR) 7.96, 95% confidence interval (CI) 2.77‒22.91), awakening pain (OR 3.14, 95% CI 4.15–6.82), duration of headache >1 week (OR 10.59, 95% CI 2.9–38.7), fever (OR 6.01, 95% CI 2.07–17.46), worst headache ever (OR 12.95, 95% CI 5.69–29.45), alteration of consciousness (OR 13.55, 95% CI 2.07‒88.88), and localizing neurological deficit (OR 5.28, 95% CI 1.6‒17.46). A score ≥3 out of 10 points of the clinical prediction score was likely to identify a serious intracranial cause of acute NTH with a sensitivity and specificity of 87.50% (95% CI 78.73–93.59%) and 87.70% (95% CI 83.90–90.89%), respectively. The area under the curve was 0.933.

**Conclusion:**

Abrupt onset, awakening pain, duration of headache >1 week, fever, worst headache ever, alteration of consciousness, and localizing neurological deficit were the significant predictive factors for serious intracranial cause of acute NTH.

## 1. Introduction

Acute nontraumatic headache (NTH) is a common chief complaint, and 8–13% of patients have serious intracranial causes at the emergency department (ED) [1–3]. The emergency physician (EP) makes a diagnosis by evaluating the clinical presentation, physical examination, and red flag signs to choose the appropriate further investigation and treatment. Therefore, the challenge for the EP is to evaluate and diagnose a life-threatening headache because high mortality rates and severe disabilities were reported [4]. Errors in diagnosing serious intracranial cause usually occur at the ED [4]. Serious intracranial causes that the EP must not misdiagnose are acute subarachnoid hemorrhage (SAH), acute ischemic stroke, acute intracerebral hemorrhage (ICH), neoplasm, intracranial infection, cerebral venous sinus thrombosis, hypertensive encephalopathy, arteriovenous malformation (AVM), hydrocephalus, and giant cell arteritis [1, 2, 5]. The aim of this study was to identify the predictive factors and develop a clinical prediction score to identify serious intracranial cause in acute NTH patients.

## 2. Methods

### 2.1. Study Design and Setting

A retrospective chart review study was conducted at the ED of Songklanagarind Hospital, which is a tertiary university hospital in southern Thailand. The data were collected from January 2013 to June 2018 and followed the methods of Tantarattanapong et al. [6]. The inclusion criteria were (1) age ≥15 years and visited the ED with the chief complaint of acute NTH and (2) the final diagnosis was a serious intracranial cause that included acute SAH, acute ischemic stroke, acute ICH, neoplasm, intracranial infection, cerebral venous sinus thrombosis, hypertensive encephalopathy, AVM, hydrocephalus, or giant cell arteritis. The definition for diagnosis of acute NTH was according to the International Classification of Headache Disorders, 3^rd^ edition (beta version) [7]. The exclusion criteria were (1) patients diagnosed as serious intracranial cause who were referred from other hospitals, (2) pregnancy, (3) history of traumatic brain injury within the previous 30 days [8], (4) previous intracranial pathology, (5) incomplete medical records, and (6) lost to follow-up.

The study was approved by the Institutional Ethics Committee Board of the Faculty of Medicine at Prince of Songkla University. Informed consent was waived according to our institutional review board protocol because the research presented no more than minimal risk to the participants and did not involve procedures for which written consent is normally required outside the research context. All research information was kept confidential and was accessed only by the researcher and the assistant.

When the patients presented at the ED, the EP evaluated the history, physical examination, and used red flag signs to classify the risk for diagnosis between primary and secondary headache. The red flag signs were age >50 years, abrupt or sudden onset, positional provocation, systemic symptoms (fever and weight loss), secondary risk factors (i.e., HIV infection and malignancy), neurological symptoms (i.e., alteration of consciousness and focal neurological deficit), and papilledema [9]. The provisional diagnosis was made by the EP after complete history taking and physical examination. The patients who had red flag signs needed further investigations such as neuroimaging or lumbar puncture as indicated. The results of imaging were reported by the radiologist. If the patients had a serious intracranial cause, the EP consulted the specialists (i.e., internist, neuromedical, and neurosurgical physicians) to confirm the final diagnosis and treatment. If the patients did not have red flag signs, they were followed up at the medicine outpatient department to confirm a final diagnosis.

### 2.2. Data Collection

The collected data from the medical records included the patient baseline characteristics, history taking, physical examination, red flag signs, provisional diagnosis, investigations, and final diagnosis. The patients were then categorized into serious intracranial headache and nonserious intracranial headache groups.

### 2.3. Outcome Measurements

The primary outcome was identification of the predictive factors of serious intracranial cause in acute NTH patients. The secondary outcome was to develop a clinical prediction score to identify serious intracranial cause in acute NTH patients.

### 2.4. Statistical Analysis

The *R* software, version 3.2.2, was used for the statistical analysis. The median values were calculated for continuous variables, while percentages were calculated for discrete variables. A bivariate analysis was used to analyze the baseline characteristics, clinical presentation, physical examination, and red flag signs. The data were compared between serious and nonserious intracranial headache. Continuous variables were compared using the Mann–Whitney *U* test. Categorical variables were compared using the *χ*^2^ or Fisher's exact test. A binary multivariate logistic regression was used to identify the significant predictive factors associated with serious intracranial headache. Statistical significance was defined as *p* ≤ 0.05, and the results are presented as odds ratio (OR) with 95% confidence interval (CI). The clinical predictive score for serious intracranial cause was analyzed by coefficients from the multivariate logistic regression to plot a receiver operating characteristic (ROC) curve. A plot of sensitivity vs. 1 − specificity gave rise to the ROC curve.

## 3. Results

**Figure 1:**
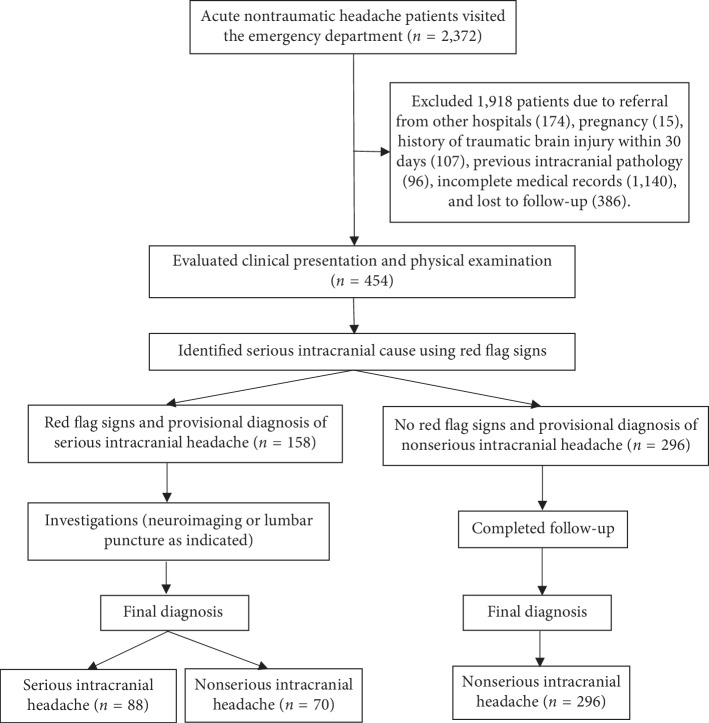
Flow chart of this study.

A total of 2,372 patients visited the ED with the chief complaint of acute NTH. A total of 1,918 patients were excluded, and 454 patients met the inclusion criteria (Figure 1). One hundred fifty-eight (34.80%) patients had red flag signs, and 88 (19.38%) patients had a final diagnosis of serious intracranial headache.

The baseline characteristics, clinical presentations, and physical examinations are shown in Table 1. Serious intracranial headache had a higher rate of HIV infection (8% vs. 1.4%, *p*=0.003) and malignancy (15.9% vs. 5.7%, *p*=0.003) compared to nonserious headache. Furthermore, 2.3% and 3.4% of serious intracranial headache patients had taken prednisolone and hormone therapy, respectively.

In the serious intracranial headache group, the clinical presentations of gradual onset, abrupt onset, duration of headache >1 week, awakening pain, worst headache ever, neck pain, nausea/vomiting, fever, and seizure presented in 62.5%, 37.5%, 25%, 47.7%, 87.5%, 33%, 64.8%, 25%, and 8% of the cases, respectively, with statistical significance.

On physical examination, the patients in the serious intracranial headache group had abnormal neurological examination more than the patients in the nonserious intracranial headache group: altered consciousness (6.8% vs. 0.3%, *p* < 0.001), localizing neurological deficit (22.7% vs. 3.6%, *p* < 0.001), and stiffness of neck (17% vs. 1.1%, *p* < 0.001) (Table 1).

In 454 patients with acute nontraumatic headache, 158 (34.8%) patients had red flag signs and needed further investigations. The significant red flag signs in serious intracranial headache were abrupt onset (37.5%), systemic symptoms (25%), secondary risk factors (23.9%), and neurological deficit (33%) (Table 2). For serious intracranial headache, 88 patients had red flag signs and the investigations performed were CT (100%), CTA (18.2%), MRI (26.1%), and lumbar puncture (29.5%). The results of the investigations are shown in Table 2. In the nonserious intracranial headache group of 366 patients, 70 (19.12%) patients had red flag signs and needed further investigations (Table 2) and the final diagnosis was nonserious intracranial headache.

The provisional diagnosis of serious intracranial causes by the EP was in 84 patients, and a misdiagnosis occurred in 4 (4.8%) patients who had brain metastasis, subacute subdural hemorrhage, acute ischemic stroke, and giant call arteritis. The top five final diagnoses of serious intracranial causes were neoplasm, intracranial infection, ICH, acute SAH, and ischemic stroke (Table 3).

Multivariate analysis revealed that the significant predictive factors to identify serious intracranial causes in acute NTH patients were abrupt onset, awakening pain, duration of headache >1 week, fever, worst headache ever, alteration of consciousness, and localizing neurological deficit (Table 4).

**Figure 2:**
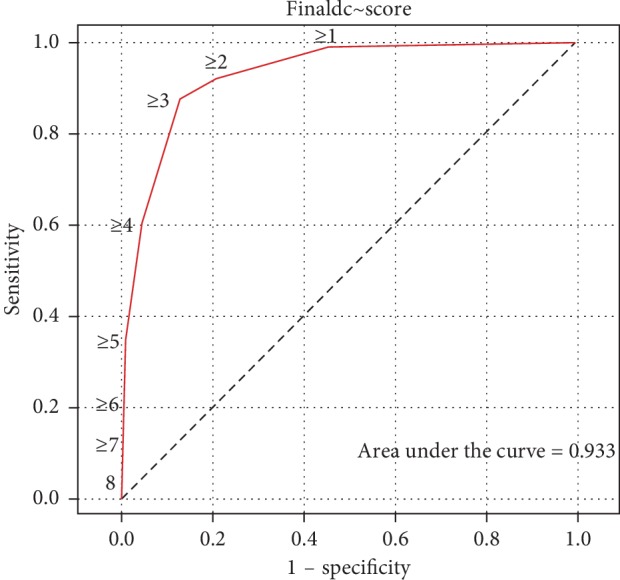
Receiver operating characteristic curve derived from the clinical prediction score to diagnose serious intracranial causes in acute nontraumatic headache.

The significant predictive factors were developed into a clinical prediction score for serious intracranial cause (Table 5). A score <3 points means the patient is less likely to have a serious intracranial cause, and a score ≥3 points means the patient is likely to have a serious intracranial cause. The sensitivity and specificity were 87.50% (95% CI 78.73–93.59%) and 87.70% (95% CI 83.90–90.89%), respectively. From Figure 2, the area under the curve is 0.933.

## 4. Discussion

The incidence of serious intracranial cause in this study was 19.38%, which was higher than that reported in other studies (8.9–13%) [1–3]. The common causes of serious intracranial causes in acute NTH were neoplasm and intracranial infection, which were different from other studies that reported acute SAH as the most common cause [1–3].

The significant factors of serious intracranial causes in acute NTH in this study were the same factors as the red flag signs, which consisted of abrupt onset, systemic symptoms, secondary risk factors, and neurological deficit. These factors were associated with the most common etiology of this study. From a study by Lamont, the red flag signs, which consisted of papilledema, drowsiness, confusion, memory impairment or loss of consciousness, and paralysis, were quite similar to this study [10].

It is interesting to note that 95% had at least two of the four symptoms of headache, fever, neck stiffness, and altered mental status, which could be diagnosed as meningitis from the study by van de Beek [11]. According to a study by Pfund, if the patient had duration of headache from one week to a month and severe (worst) headache pain, it had a high diagnostic yield for the diagnosis of serious intracranial neoplasm [12]. From these two studies, the associated symptoms were quite similar to the multivariate analysis by logistic regression in this study, which demonstrated that fever (OR 6.01, 95% CI 2.07–17.46), alteration of consciousness (OR 13.55, 95% CI 2.07–88.88), duration of headache >1 week (OR 10.59, 95% CI 2.9–38.7), and severe (worst) headache pain (OR 12.95, 95% CI 5.69–29.45) were the significant factors of serious intracranial cause in acute NTH patients.

Abrupt onset (OR 7.96, 95% CI 2.77–22.91), alteration of consciousness (OR 13.55, 95% CI 2.07–88.88), and localizing neurological deficit (OR 5.28, 95% CI 1.6–17.46) were the same significant predictive factors to diagnose serious intracranial cause as Locker [3]. The worst headache ever (OR 12.95, 95% CI 5.69–29.45) was the significant factor in a previous study, which demonstrated an association with a diagnosis of acute SAH (OR 76.5, 95% CI 6.0–982.9) [9].

In clinical practice, diagnostic algorithms have been structured for four clinical scenarios at the ED. Scenario 1 aims to include acute SAH, scenario 2 aims to include the expression of intracranial infection, scenario 3 aims to include intracranial neoplasm or temporal arteritis, and scenario 4 aims to include benign headache [13]. In scenarios 1–3, the clinical presentations are the same as the clinical presentation scoring system in this study to diagnose a serious intracranial cause. From a study by Grimaldi et al., 18 of 77 patients in scenarios 1, 2, and 3 had malignant headache [8].

This study developed a clinical prediction score to identify serious intracranial cause. Plots of sensitivity versus 1-specificity had a sensitivity and specificity of 87.50% (95% CI 78.73–93.59%) and 87.70% (95% CI 83.90–90.89%), respectively. The area under the curve was 0.933, which means this study had good overall test accuracy because a range between 0.9 and 1.0 indicates that the cutoff point to diagnosis serious intracranial cause is excellent. The cutoff point at 3 can divide the patients into two groups [14]. If the score is <3 of 10 points, it means the patient is less likely to have a serious intracranial cause, and a score ≥3 of 10 points means the patient is likely to have a serious intracranial cause.

The clinical prediction score in this study was developed from significant factors of the red flag signs. This practical scoring system facilitated the EP, so as to categorize patients as either serious or nonserious intracranial causes of acute NTH patients. Then, the EP made it easy to decide whether further investigations were required. However, the clinical prediction score, for serious intracranial causes, of this study had limitations, firstly because of a lower sensitivity than other studies [3, 13]. Additionally, an important bias in this study was the retrospective design, wherein the majority of the nonserious intracranial causes for patients were not completely investigated, such as imaging or lumbar puncture. Because of this, final diagnosis of some patients, in this group, was not fully established. Therefore, a well-designed prospective study should be conducted to overcome this limitation.

## 5. Conclusions

The EP obtains patient information from careful history taking and a physical examination. The EP should consider a diagnosis of serious intracranial cause in acute NTH patients who present with abrupt onset, duration of headache >1 week, awakening pain, fever, worst headache ever, alteration of consciousness, and localizing neurological deficit.

## Figures and Tables

**Table 1:** Baseline characteristics, clinical presentation, and physical examination.

Characteristic	Nonserious intracranial headache (366 patients)	Serious intracranial headache (88 patients)	*p* value
Age, years, median (IQR)	54.5 (42, 70)	51 (40, 64.2)	0.157
Male	131 (35.8)	41 (46.6)	0.08

*Comorbidity*			
Diabetic mellitus	42 (11.5)	12 (13.6)	0.705
Hypertension	118 (32.2)	28 (31.8)	1
Hyperlipidemia	84 (23)	18 (20.5)	0.718
Atrial fibrillation	12 (3.3)	1 (1.1)	0.478
Ischemic heart disease	17 (4.6)	3 (3.4)	0.777
Cerebrovascular disease	29 (7.9)	8 (9.1)	0.887
HIV infection	5 (1.4)	7 (8)	0.003
Malignancy	21 (5.7)	14 (15.9)	0.003

*Current medication*			
ASA	46 (12.6)	7 (8)	0.305
Clopidogrel	3 (0.8)	0 (0)	1
Anticoagulant	11 (3)	4 (4.5)	0.505
Prednisolone	0 (0)	2 (2.3)	0.037
Hormonal therapy	1 (0.3)	3 (3.4)	0.024

*Clinical presentation*			
Gradual onset	308 (84.2)	55 (62.5)	<0.001
Abrupt onset	58 (15.8)	33 (37.5)	<0.001
Duration of headache >1 week	34 (9.3)	22 (25)	<0.001
Occipital pain	60 (16.4)	21 (23.9)	0.137
Pain score	7 (5, 8)	10 (8, 10)	<0.001
Awakening pain	41 (11.2)	42 (47.7)	<0.001
Worst headache ever	61 (16.7)	77 (87.5)	<0.001
Neck pain	61 (16.7)	29 (33)	<0.001
Nausea/vomiting	149 (40.7)	57 (64.8)	<0.001
Fever	26 (7.1)	22 (25)	<0.001
Positional provocation	5 (1.4)	4 (4.5)	0.076
Seizure	5 (1.4)	7 (8)	0.003

*Physical examination*			
BT, °C, median (IQR)	36.6 (36.2, 36.9)	36.8 (36.2, 37.3)	0.008
SBP, mmHg, median (IQR)	144 (128, 161)	140.5 (127, 162)	0.621
PR, bpm, median (IQR)	76 (68, 88)	77 (67.5, 88.2)	0.89
RR, breath/min, median (IQR)	20 (20, 24)	24 (20, 24)	0.018
SpO_2_, %, median (IQR)	99 (98, 100)	99 (97, 100)	0.006
Altered consciousness	1 (0.3)	6 (6.8)	<0.001
Localizing neurological deficit	13 (3.6)	20 (22.7)	<0.001
Stiffness of neck	4 (1.1)	15 (17)	<0.001

Data are presented as *n* (%) unless indicated otherwise. *p* values <0.05 are statistically significant. IQR = interquartile range; HIV = human immunodeficiency virus; ASA = aspirin; BT = body temperature; SBP = systolic blood pressure; PR = pulse rate; bpm = beats per minute; SpO_2_ = oxygen saturation.

**Table 2:** Comparison of red flag signs and investigations.

	Nonserious intracranial headache (366 patients)	Serious intracranial headache (88 patients)	*p* value
*Red flag signs*			
Age >50 years	231 (63.1)	48 (54.5)	0.174
Abrupt onset	58 (15.8)	33 (37.5)	<0.001
Positional provocation	5 (1.4)	4 (4.5)	0.076
Systemic symptoms	26 (7.1)	22 (25)	<0.001
Secondary risk factors	26 (7.1)	21 (23.9)	<0.001
Neurological deficit	13 (3.6)	29 (33)	<0.001

*Investigations*			
Performed CT brain	70 (19.1)	88 (100)	<0.001

*CT brain results*			
Subarachnoid hemorrhage	0 (0)	12 (13.6)	<0.001
Intracerebral hemorrhage	0 (0)	15 (17.0)	<0.001
Infarction	0 (0)	6 (6.8)	<0.001
Tumor with/without complications	0 (0)	21 (23.9)	<0.001
Venous sinus thrombosis	0 (0)	4 (4.5)	0.001
Hydrocephalus	0 (0)	1 (1.1)	0.194
Brain abscess	0 (0)	1 (1.1)	0.194
Within normal limits	70 (19.1)	28 (31.8)	0.014
Performed CTA	0 (0)	16 (18.2)	<0.001

*CTA results*			
AVM	0 (0)	6 (6.8)	<0.001
Aneurysm	0 (0)	7 (8)	<0.001
Within normal limits	0 (0)	3 (3.4)	0.007
Performed MRI	0 (0)	23 (26.1)	<0.001

*MRI results*			
Leptomeningeal enhancement	0 (0)	1 (1.1)	0.194
Focal meningoencephalitis	0 (0)	1 (1.1)	0.194
Brain tumor/metastasis	0 (0)	17 (19.3)	<0.001
Cerebral infarction	0 (0)	2 (2.3)	0.037
Venous sinus thrombosis	0 (0)	1 (1.1)	0.194
AVM	0 (0)	1 (1.1)	0.194
Performed lumbar puncture	5 (1.4)	26 (29.5)	<0.001

*CSF findings*			
Bacterial meningitis	0 (0)	2 (2.3)	0.037
Viral meningitis	0 (0)	12 (13.6)	<0.001
Fungal meningitis	0 (0)	6 (6.8)	<0.001
Eosinophilic meningitis	0 (0)	1 (1.1)	0.194
Blood	0 (0)	1 (1.1)	0.194
Normal profile	5 (1.4)	4 (4.5)	0.076

*Pathogens*			
Beta Streptococcus group B	0	1	
*Pseudomonas aeruginosa*	0	1	
*Cryptococcus neoformans*	0	5	
Human herpes virus	0	2	

Data are presented as *n* (%) unless indicated otherwise. *p* values <0.05 are statistically significant. CT = computed tomography; CTA = computed tomographic angiography; AVM = arteriovenous malformation; MRI = magnetic resonance imaging.

**Table 3:** Final diagnosis of serious intracranial cause of acute nontraumatic headache.

Final diagnosis	No. of patients (%)
Neoplasm	23 (26.1)
Intracranial infection	22 (25.0)
Intracerebral hemorrhage	15 (17.0)
Acute subarachnoid hemorrhage	13 (14.8)
Ischemic stroke	6 (6.8)
Cerebral venous sinus thrombosis	5 (5.7)
Hypertensive encephalopathy	1 (1.1)
Arteriovenous malformation	1 (1.1)
Hydrocephalus	1 (1.1)
Giant cell arteritis	1 (1.1)
Total	88 (100)

**Table 4:** Multivariable logistic regression model for the predictive factors of serious intracranial cause in acute nontraumatic headache.

Variables	Crude OR (95% CI)	Adjusted OR (95% CI)	*p* value
Abrupt onset	3.19 (1.9–5.33)	7.96 (2.77–22.91)	<0.001
Awakening pain	7.24 (4.26–12.29)	3.14 (4.15–6.82)	0.004
Duration >1 week	4.06 (2.12–7.78)	10.59 (2.9–38.7)	<0.001
Fever	4.36 (2.33–8.15)	6.01 (2.07–17.46)	<0.001
Worst headache ever	35 (17.57–69.71)	12.95 (5.69–29.45)	<0.001
Alteration of consciousness	40.33 (11.74–138.6)	13.55 (2.07–88.88)	0.002
Localizing neurological deficit	8.36 (3.96–17.63)	5.28 (1.6–17.46)	0.019

*p* values <0.05 are statistically significant. OR = odds ratio; CI = confidence interval.

**Table 5:** Clinical prediction scores for serious intracranial cause in acute nontraumatic headache.

Predictive factors	Points
Abrupt onset	1
Awakening pain	1
Duration >1 week	1
Fever	1
Worst headache ever	2
Alteration of consciousness	3
Localizing neurological deficit	1
Total	10

Note: a score of ≥3 points is predictive of serious intracranial cause.

## Data Availability

All data are available within the article.
